# Editorial: Ovarian Stimulation, Endocrine Responses and Impact Factors Affecting the Outcome of IVF Treatment

**DOI:** 10.3389/fendo.2022.857089

**Published:** 2022-04-14

**Authors:** Barbara Lawrenz, Human Fatemi

**Affiliations:** ^1^IVF Department, ART Fertility Clinic, Abu Dhabi, United Arab Emirates; ^2^Obstetrical Department, Women’s University Hospital Tübingen, Tübingen, Germany

**Keywords:** ovarian stimulation, assisted reproductive techniques, final oocyte maturation, endocrine evaluation, Oocyte yield

## Introduction

Assisted reproductive treatments are a tool to overcome infertility and are used worldwide to treat patients, suffering from this condition. Pregnancy chances have increased over the last decades due to improvement of the techniques for ovarian stimulation, although, there is still a lack of treatment individualization according to the patients’ characteristics.

## The Main Points of Individual Contributions

This series includes 11 papers, all original research articles, which are referred to in the order, which would apply for basic assessment, evaluation and treatment of the infertile couple.

Primary assessment of the female partner involves basic characteristics like recording of age, body mass index (BMI) and cycle history. Usually with this information, patients with a polycystic ovarian syndrome (PCOS) are easy to identify and, as Insulin resistance (IR) is a common metabolic problem in these patients (1), this metabolic condition can be confirmed or exclude by performing an oral glucose tolerance test (OGTT). However, also lean patients with regular cycles might have IR (2), impacting the outcome of an ART treatment. In their prospective, observational study, Wang et al. subjected lean, non-PCOS patients to an OGTT and compared the stimulation outcomes between patient with and without IR. In their study, IR was associated with a slower response to the ovarian stimulation, a poorer oocyte maturation and a decreased proportion of freezable embryos, as compared to non-IR women. Further on, lean patients with IR had a higher prevalence of subclinical hypothyroidism.

Prolactin (PRL) is one of the hormones evaluated usually in the primary assessment, as elevated PRL levels can cause cycle irregularities and anovulation (3). However, in cases with mildly elevated PRL levels, the question remains whether a treatment is indicated to lower the PRL levels prior to initiating an IVF cycle. Zhang et al. evaluated retrospectively the influence of basal PRL levels on the pregnancy outcomes in patients, undergoing ovarian stimulation in a long GnRH-agonist protocol due to tubal or male factor infertility. Based on their findings, the authors concluded that in patients with a basal PRL level within the range of 0–50 ng/mL, higher PRL levels were associated with higher numbers of oocytes, mature oocytes, zygotes, and embryos. Also, cumulative clinical pregnancy rate (CPR) and live birth rate (LBR) increased with increasing PRL levels. These data point to the fact, that for patients with an asymptomatic, mild hyperprolactinemia, planning to undergo an IVF/ICSI treatment, the PRL serum level may be not suppressed to an extremely low level, provided that organic lesions were excluded.

Success rates can be severely impacted by uterine abnormalities, with adenomyosis being one of them (4). It is well known, that pretreatment with GnRH agonists can reduce the size of the adenomyotic areal and therefore reduce the negative impact of the adenomyosis on the IVF outcome. However, the benefit of a long term pretreatment with GnRH agonists, prior to an ovarian stimulation for IVF/ICSI in a long GnRH agonist protocol is discussed controversially and the data of Chen et al. do not support GnRH agonist pretreatment in this setting.

The ovarian reserve parameters Anti-Muellerian-Hormone (AMH) and Antral Follicle Count (AFC) are together with patients’ characteristics like age, BMI and the outcome of a possible previously performed ovarian stimulation treatment, the basis for deciding on the gonadotropin dosage (5). The most commonly used protocols are the GnRH (Gonadotropin-Releasing-Hormone)- agonist and GnRH-antagonist protocol. In 2020, a guideline was published by the ESHRE group on ovarian stimulation to summarize the available data (6). Huang et al. evaluated in a retrospective study, whether AMH remains a reliable predictor for the outcome not only in commonly used ovarian stimulation protocols, but also in progestin-primed ovarian stimulation protocols. According to their analysis, AMH correlates well also in this kind of protocol, independent of the dose of medroxyprogesterone acetate, used to prevent ovulation.

The number of retrieved/mature oocytes is crucial for the success (7) and treatment of poor responder patients (8) and remains a challenge for the reproductive medicine specialist. In a retrospective approach, Orvieto et al. analyzed the treatment of patients, who have had – in a conventional stimulation protocol - a previous poor response and who were treated with a combined Stop GnRH-antagonist protocol subsequently. With this approach, a significantly higher numbers of oocytes were retrieved, as well as higher numbers of embryos transferred, as compared to their previous IVF attempt.

During ovarian stimulation, serum FSH reflects the *in vivo* serum FSH levels to which the ovaries are exposed (9). In the search of tools for early individualization of the stimulation protocol, serum delta FSH levels between D6 of gonadotrophin use and basal serum FSH as well as between D6 of gonadotrophin use and D1 of gonadotrophin use have been investigated by Hu et al., in order to predict ovarian response.

Choosing the “correct” timing for the administration of the medication for final oocyte maturation (so called “trigger”) is crucial for the retrieval of mature oocytes. The size of the follicles and the measurement of estradiol (E2) are the parameters commonly used to determine the optimal time point. However, due to the multifollicular growth of follicles of varying size, serum E2 levels are supraphysiological and therefore might render E2-measurement unreliable as a determinant of oocyte maturity. To add a diagnostic tool for this decision process, the paper of Lawrenz et al. evaluated the role of Inhibin A, which is only released from a follicle size of 12mm and beyond, as a parameter of oocyte maturity.

Human Choriongonadotropin (hCG) was long considered to be the “gold standard” for final oocyte maturation, however in high responder patients or in oocyte donation patients, the administration of GnRH (Gonadotropin-Releasing-Hormone) – agonist is meanwhile standard to avoid ovarian hyperstimulation syndrome (OHSS) (9). In seldom cases, administration of GnRH-agonist fails to be effective and no oocytes will be retrieved, which leads to disappointment and possibly loss in trust. The study of Cozzolino et al. evaluated the reliability of a urinary LH-self test 12 hours after administration of the GnRH-agonist trigger in oocyte donation patients. As a positive urinary LH test after GnRH-agonist trigger proved to be a reliable tool for retrieving mature oocytes, it could be used in the monitoring process to detect errors in the administration and/or inadequate responses to the trigger and therefore improve the outcome. Due to their different mode of action, the administration of various kinds of “trigger” medications (hCG, GnRH-agonist and kisspeptin) result in altered endocrine profiles. These profiles have been investigated by Abbara et al., revealing distinct differences, which should be taken into account when individualizing treatment protocols according to patients’ characteristics.

In a secondary analysis from previously published data, Benmachiche et al. evaluated the correlation of preovulatory LH levels in a GnRH-antagonist protocol and the use of a GnRH-agonist for final oocyte maturation, and the cycle outcome in cycles with fresh embryo transfer and the use of a modified luteal phase support. According to their data, low pre-ovulatory LH levels might reduce the chance for a pregnancy, but further studies are warranted.

Progesterone measurement and the impact of the progesterone levels in the luteal phase on the ART outcome is a “hot-topic” and discussed controversially. Besides progesterone, also 17-OH progesterone (17-OH P4) is produced by the corpus luteum (CL) and 17-OH P4 levels are not influenced by luteal phase support. In order to evaluate whether 17-OH P4 would give a better parameter for the monitoring of the luteal phase, Thomsen et al. evaluated prospectively the correlation with the ART outcome.

## Synthesis and Conclusion

This “Research Topic” includes papers which evaluate the impact/meaningfulness of hormonal parameters “deviating from the well-trodden path” of commonly used hormones and may open new insights into diagnostic and treatment options.

## Author Contributions

BL: writing the editorial HF: reviewing the editorial. All authors contributed to the article and approved the submitted version.

## Conflict of Interest

The authors declare that the research was conducted in the absence of any commercial or financial relationships that could be construed as a potential conflict of interest.

## Publisher’s Note

All claims expressed in this article are solely those of the authors and do not necessarily represent those of their affiliated organizations, or those of the publisher, the editors and the reviewers. Any product that may be evaluated in this article, or claim that may be made by its manufacturer, is not guaranteed or endorsed by the publisher.
